# Unsupervised Performance of the CogState Brief Battery in the Brain Health Registry: Implications for Detecting Cognitive Decline

**DOI:** 10.14283/jpad.2021.68

**Published:** 2022

**Authors:** T. Banh, C. Jin, J. Neuhaus, R.S. Mackin, P. Maruff, N. Stricker, M.W. Weiner, R.L. Nosheny

**Affiliations:** 1. University of California San Francisco, USA;; 2. Cogstate Ltd, USA;; 3. Mayo Clinic College of Medicine and Science, USA

**Keywords:** Cognitive decline, neuropsychology, Brain Health Registry

## Abstract

**INTRODUCTION:**

The feasibility and validity of unsupervised, longitudinal brief computerized cognitive batteries is unknown.

**METHODS:**

Participants aged 56–90 (N = 19476) from the Brain Health Registry (BHR) completed the CogState Brief Battery (CBB) at 6-month intervals over a period of 5 years. We used linear mixed-effects models to assess whether cross-sectional and longitudinal performance on CBB within BHR was associated with demographic and cognitive characteristics. We also defined a group of CBB decliners based on subject-specific slopes and estimated associations between decliner status and participant characteristics.

**RESULTS:**

We found weak associations between longitudinal change in CBB and participant characteristics. Cross-sectional CBB scores were significantly associated with participant characteristics such as age, gender, ethnicity, self-reported disease status, and memory concern. CBB decliners were more likely to self-report mild cognitive impairment (MCI) and memory concerns.

**DISCUSSION:**

Cross-sectional, remote CBB shows evidence of construct validity, but our results suggest that longitudinal assessment may not provide additional value for identifying those at risk for and with cognitive impairment.

## Introduction

The biological changes that characterize Alzheimer’s disease (AD) begin up to 30 years before dementia is clinically diagnosed (1). This long pre-dementia period provides an opportunity for interventions designed to slow or even stall AD progression. However, within this pre-dementia stage, careful neuropsychological assessment does show a subtle but progressive worsening of cognition, particularly in aspects of memory and learning. Therefore, greater understanding of the nature and magnitude of such decline across the pre-dementia period will both increase understanding of AD brain behavior models and also provide a basis for assessing the effectiveness of any interventions (2, 3). To date, most descriptions of cognitive changes that occur in early AD come from neuropsychological assessments conducted in controlled environments by appropriately trained raters. However, where cognition is assessed to provide input into disease risk algorithms, the use of highly controlled but unsupervised cognitive assessment, often delivered though web based computer controlled interfaces, has emerged as an efficient and feasible method for obtaining valid and reliable cognitive data (4–9).

Compared to the standard supervised administration of neuropsychological tests, the remote and unsupervised use of computer based cognitive tests may also be better suited for the longitudinal monitoring of large cohorts of older adults. The advantages of a remote, computerized approach include rapid recruitment, less cost, reduction in administrator bias, automated scoring, and increased generalizability due to a possible large sample (10–12). Allowing individuals to take their retests at home may increase compliance and retention through the reduction burden and travel costs. Remote assessments could increase the power of prospective studies to observe their phenomena of interest because their low demands could allow more frequent reassessments, and shorter retest intervals. Finally, the reduced demands required for home based cognitive assessments could provide a basis for improving study designs to be more inclusive. However, while there has now been a good deal of research aimed at understanding the usability, acceptability, and validity of unsupervised cognitive testing within cross-sectional designs, there is comparatively less information about these important characteristics for studies with longitudinal designs, especially in older adults. Although the clinical utility of unsupervised neuropsychological tests is an active area of research (9, 13, 14), most studies to date have focused on cross-sectional test performance (4). Few studies have addressed whether longitudinal testing can predict cognitive decline (5, 14), especially in large samples of older adults.

The Brain Health Registry (BHR), established in 2014, is a large online registry and cohort with longitudinal, cognitive, demographic, and clinical data from over 70,000 participants (15). The CogState Brief Battery (CBB) is a computerized cognitive assessment that is administered to participants enrolled in BHR. Clinical pathological studies of early AD using the CBB in supervised contexts have shown that the learning and working memory assessments in this battery are sensitive to deterioration in cognition over time in individuals who carry biological changes indicative of AD (3, 16, 17). Prior research has shown cross-sectional assessment of CBB in BHR to be associated with advanced age and self-report of cognitive impairments (4). Other studies have shown that the CBB can be optimized for use in remote unsupervised contexts and retain its sensitivity to AD related clinical and biological characteristics. However, the extent to which the CBB can be applied repeatedly to understand age and dementia related cognitive change, in remote and unsupervised contexts is not understood. Therefore the aim of this study was to understand the nature and magnitude of cognitive change, determined from longitudinal application of the CBB in the BHR, and then determine whether this was associated with demographic and other clinical characteristics of the sample. The hypothesis was that in older adults and those with self-reported cognitive impairments, memory concerns, and family history of AD would perform worse over time on CBB compared to those without.

## Methods

### Participants

The BHR was established by the University of California, San Francisco and is reviewed by an institutional review board. Enrollment began in March 2014 and is ongoing. Details of the study design and sampling procedures have been previously published (15).

The present analysis includes N = 19476 participants aged 56 to 90 years who completed online, self-report questionnaires on a variety of demographic factors (age, gender, education level, race, and ethnicity), medical history (self-reported mild cognitive impairment (MCI), AD, and dementia, memory concern, family history of AD), and an unsupervised version of the CBB. Participants self-administer the test through the BHR website. Before each subtest, participants complete a practice session to get acquainted. Participants are invited by email to complete the CBB at 6-month intervals.

**Figure 1.**
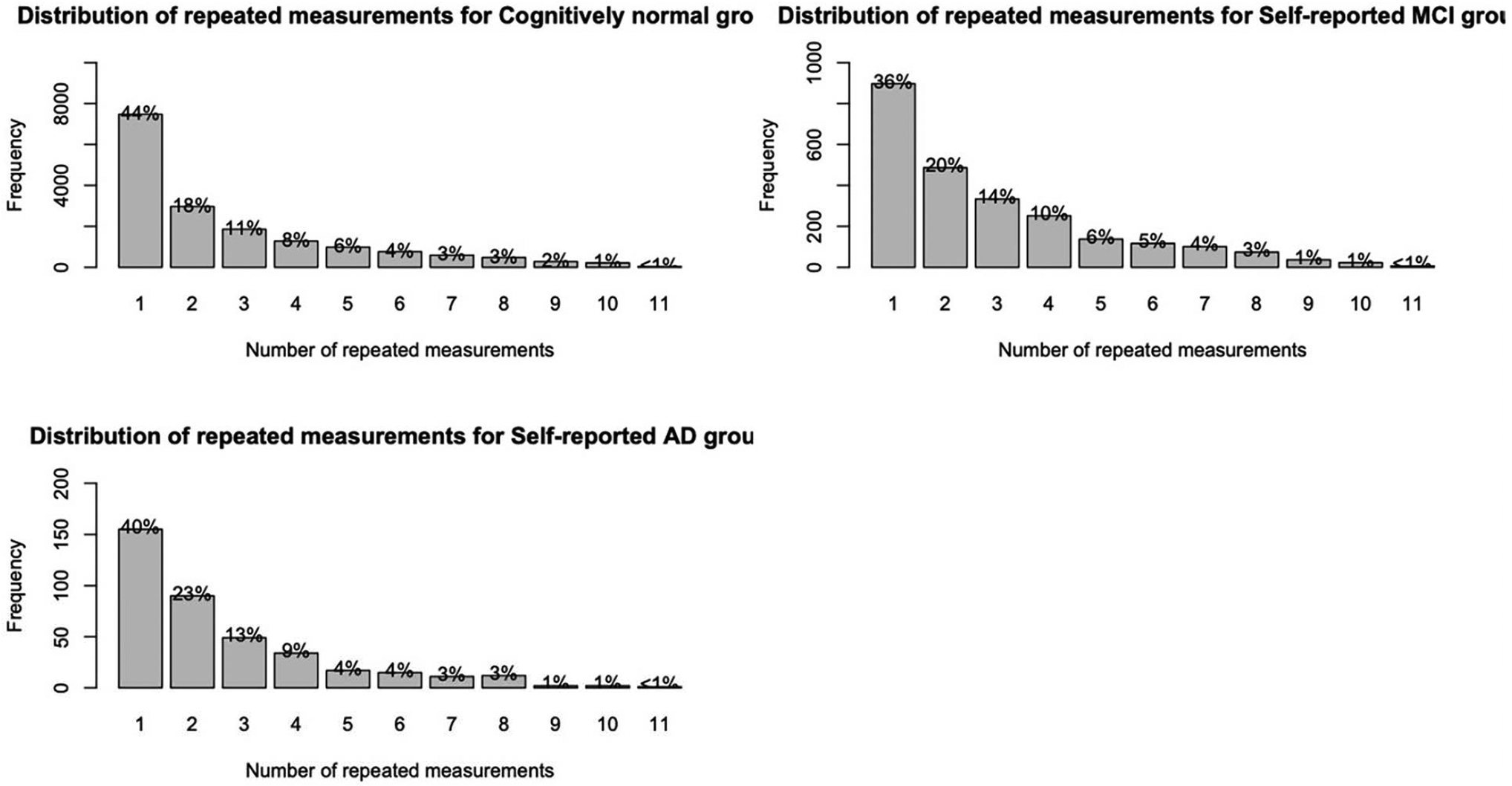
Distribution of repeated measurements stratified by self-reported disease status

Table 1 contains participant information. Of the 19476 participants, 2459 self-reported an MCI diagnosis, and 388 self-reported an AD diagnosis. Figure 1 displays the distribution of repeated measurements for the sample stratified by self-reported disease status.

### CogState Brief Battery

The CBB is a computerized cognitive assessment that has been previously validated with repeated assessment in healthy participants (18–20) and those with mild cognitive impairment (MCI) or early AD (21–23) in both supervised (16, 24, 25) and unsupervised (8, 26, 27) contexts. The CBB consists of four subtests. The Detection (DET) task tests information-processing speed, attention and motor speed. Participants engage in a simple reaction time (RT) paradigm where they must respond as soon as the presented card changes. The Identification (IDN) test measures visual attention and is a choice RT paradigm in which participants must decide as quickly as possible whether the presented card is red. The One-Card Learning (OCL) test measures visual learning and memory and is based on a pattern separation paradigm in which participants must decide whether a presented card has been seen previously in the task; some of the cards have been shown previously, some have not, and some have not been shown but are similar to those that have. The One-Back (ONB) test measures working memory and is based on the one back paradigm in which participants must decide whether the card they are looking at is the same as the card that was presented on the immediately previous trial. On each trial on each test, participants are instructed to respond Yes or No as quickly and as accurately as possible and measures of the speed and accuracy of responses are obtained for each test.

### Family History of Alzheimer’s Disease

Family history of AD was obtained from participants from BHR. From BHR’s inception to 8/28/2019, participants answered the following question: “Have you, your sibling(s), or parent(s) ever been diagnosed with Alzheimer’s Disease?”. Beginning on 8/28/2019 to 9/28/2020, the question was worded as such: “Have your children, your sibling(s), or parent(s) ever been diagnosed with Alzheimer’s Disease? Finally, from 9/28/2020 onwards, the question was worded: “Do you have any biological parents, full siblings, or biological children who have been diagnosed with Alzheimer’s Disease?”

### Subjective memory concern

Subjective memory concern was collected by asking the following question: “Are you concerned that you have a memory problem?”.

### Self-reported Alzheimer ‘s disease/mild cognitive impairment

As part of their medical history questionnaire, BHR participants were asked, “Please indicate whether you currently have or have had any of the following conditions in the past.”, where AD or MCI were possible options for participants to select. Participants could self-report having both MCI and AD, leading to overlaps.

### Decliner status

A CBB decliner was defined based on the identification of worsening performance on each CBB subtest over the study period. For the DET, IDN, and ONB subtests, the 95th percentile subject-specific slope was identified as an objective threshold for decliner status. For the OCL subtest, this cut-off was set at the 5th percentile. This methodology is consistent with that applied in previous studies of cognitive change using CBB performance in supervised contexts (2, 3).

### Statistical Analysis

For this analysis, we included all BHR participants with the following inclusion criteria: (1) age 55 years or older (2) had completed at least one CBB subtest (3) had completed all other self-report information described above. The raw data from each CBB subtest is skewed, so the data (mean RT of correct responses) were transformed using a logarithmic base 10 transformation and accuracy data (proportion correct) using an arcsine transformation (17, 19, 28). These transformations were applied by CogState. CBB data were subject to completion and integrity checks as described in previous research (29). Data that failed these checks were excluded.

Performance on each CBB subtest was modeled separately using a linear mixed-effects model (LMEM) with random intercepts and time effects. For each model, performance was modeled as a function of time (scaled to years), baseline age, gender, education level, self-reported AD diagnosis, self-reported MCI diagnosis, family history of AD, and memory concern, as well as their interactions with time. In each LMEM, the regression coefficient for a variable described the association of within-subject change in the predictor with change in the CBB outcome. An interaction term described the magnitudes of the associations of the baseline variables with time. For each CBB subtest, we fit a full model including all interactions effects and a reduced model with no interactions. We then assessed the statistical significance of the baseline variable by time interaction using a likelihood ratio test (LRT) between the full and reduced models.

In separate analyses, we used a series of multivariable binomial logistic regression models to examine associations between CBB “decliner status” and BHR variables. Statistical analyses were conducted using software R version 4.0.2 (30).

## Results

**Figure 2.**
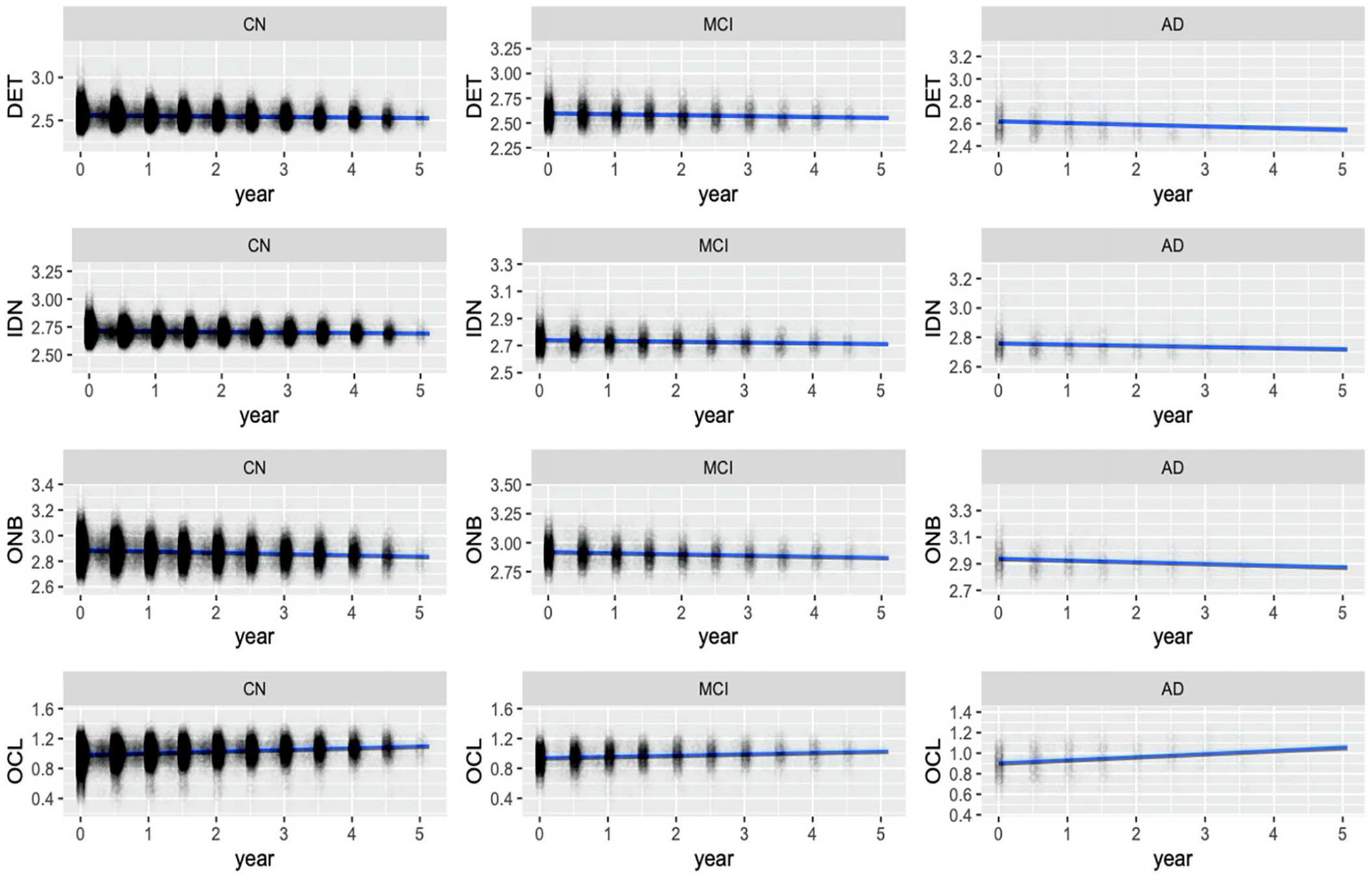
CBB performance plots stratified by self-reported disease status

Figure 2 displays CBB performance over time for the sample, stratified by self-reported disease status.

### Associations between longitudinal change in CBB and BHR variables

Parameter estimates from the LMEMs are presented in Table 2. In the OCL subtest, higher baseline age was associated with worse longitudinal performance (β = −.0003, Standard error (SE) = .00007, p < .001). Compared to cognitively normal (CN) participants, self-reported MCI participants performed worse over time on the ONB (β = .002, SE = .0007, p < .05) (positive coefficient implies worsening RT) and OCL (β = −.005, SE = .001, p < .001) subtests. Furthermore, those who reported memory concern performed worse over time on the DET subtest (β = .002, SE = .0006, p < .05). All other significant effects were baseline associations.

For each subtest, a LRT was conducted between a LMEM with interactions between time and clinical/demographic variables and a LMEM without such interactions. For the DET (χ^2^(8) = 14.57, p = .07) and IDN (χ^2^(8) = 11.72, p = .16) subtests, removing the interaction effects did not lead to better model fit. For the ONB (χ^2^(8) = 16.94, p < .05) and OCL (χ^2^(8) = 50.18, p < .01) subtests, the LRTs were significant, meaning that the models with interaction effects fit the data better.

### Associations between decliner status and BHR variables

Of the 19476 participants, 943 exceeded the DET 95% cut-off (> −.00002), 940 exceeded the IDN 95% cut-off (> −.001), 934 exceeded the ONB 95% cut-off (> −.004), and 961 were below the OCL 5% cut-off (.037). These participants were marked as decliners. Odds ratios from the logistic regression predicting decline on CBB using BHR demographic/clinical variables are presented in Table 3.

Self-reported MCI status was significantly associated with decliner status on all measures based on reaction times including DET (Odds ratio (OR) = 1.63, 95% Confidence interval (95% CI) = [1.35, 1.95]), IDN (OR = 1.45, 95% CI = [1.20, 1.76]), and ONB (OR = 1.26, 95% CI = [1.02, 1.57]) subtests, but not the OCL subtest. Furthermore, memory concern was significantly associated with decline on DET (OR = 1.15, 95% CI = [1.00, 1.34]) and ONB (OR = 1.26, 95% CI = [1.09, 1.47]) subtests. Higher baseline age was significantly associated with decline on DET (OR = 1.01, 95% CI = [1.00, 1.02]).

## Discussion

The aim of this study was to determine the value of unsupervised repeated administration of a brief computerized cognitive test battery in a large cohort of older adults. Overall, we found no associations, or very weak associations, between longitudinal change in CBB and participant characteristics, including age, self-reported MCI or AD, family history of AD, and memory concern. The strongest associations we found was that CBB decliner status, defined as those in the highest 5% of decline in a LMEM regression model, was significantly associated with self-reported MCI and memory concern. For the CBB working memory (ONB) and learning (OCL) subtests, models including interactions effects summarizing longitudinal associations performed modestly better than those including only baseline CBB performance. These mixed results suggest that remote, unsupervised, longitudinal CBB may have limited utility in helping to identify older adults with cognitive decline or early cognitive impairments in large, registry-based cohorts such as BHR.

Our first hypothesis was that older participants and those with self-reported cognitive impairments or memory concerns would perform worse on CBB over time. We found that in the OCL subtest, older participants had worse performance over time. Furthermore, in those with self-reported MCI, working memory (ONB) and learning (OCL) performance significantly decreased over time. Compared to CN participants, however, those self-reporting AD did not perform significantly different over time. Our results are consistent with research that suggests that longitudinal OCL performance may be sensitive to preclinical AD, but cross-sectional OCL may be sufficient in detecting future cognitive decline (31). When participants were categorized into CBB decline and non-decliner groups, we found that decliners were more likely to self-report MCI and report memory concern, which is consistent with prior research (16, 22). More specifically, decliners were more likely report memory concern on the DET task. This is noteworthy, considering that this is a test of attention, which may be difficult for those who report memory concern.

Taken together, our research is in agreement with a recent study on the clinical utility of longitudinal CBB, which is that longitudinal CBB only offers modest benefit compared to cross-sectional assessment in detecting cognitive impairment (13, 31). Though we did find some significant associations with repeated CBB performance, our effects were small. These effects, though statistically significant, do not imply clinical significance.

Strengths of this study include the large sample and amount of longitudinal data, combined with demographic and other self-report data. The large sample size also meant that parameters in our statistical models were statistically significant, even with very small effects. Therefore, the significance of such findings should be interpreted with caution. We further acknowledge that there was a precipitous drop in the number of repeated measurements, which also limits generalizability due to a selection bias for individuals who were highly motivated to return for repeat assessments. Finally, our sample was underrepresented for men, individuals of lower education level, and non-White/Caucasian individuals. Another limitation is our reliance on self-reported MCI and AD status, rather than clinical diagnoses, due to the inability to obtain such information within the BHR registry model. However, current research is being conducted within the registry model on a subset of participants with clinically confirmed diagnoses of AD/MCI.

In conclusion, we found that clinical utility of unsupervised, repeated assessment of CBB in a large sample of older adults is lower than expected. A similar pattern of findings was reported in the Mayo Clinic Study of Aging based predominantly on supervised CBB administrations and with clinical characterization of participants (13, 31). Interpretation of cross-sectional CBB performance may provide a simpler way to determine risk of current and future cognitive impairment given the limited added benefit of longitudinal methods.

## Figures and Tables

**Table 1.** Demographic information from BHR participants

	Full sample	Self-reported MCI group	Self-reported AD group
N	19476	2459	388
Baseline age M +/− SD *range*	66.26 +/− 6.84 [56, 90]	68.75 +/− 7.36 [56, 90]	70.36 +/− 6.85 [56, 87]
Gender (percent female)	69.5%	59.4%	51.3%
Years education M +/− SD *range*	16.32 +/− 2.39 [6, 20]	16.25 +/− 2.52 [6, 20]	16.03 +/− 2.62 [6, 20]
Race (percent White/Caucasian)	86.6%	90%	89.7%
Family history of AD (percent responded yes)	34.7%	35.2%	51.5%
Self-reported AD (percent responded yes)	2.0%	13.3%	NA
Self-reported MCI (percent responded yes)	12.6%	NA	84.0%
Subjective memory concern (percent responded yes)	65%	95.4%	92.0%
DET M +/− SD *range*	2.56 +/− .093 [2.19, 3.37]	2.59 +/− .109 [2.26, 3.28]	2.60 +/− .126 [2.39, 3.29]
IDN M +/− SD *range*	2.71 +/− .062 [2.38, 3.28]	2.73 +/− .071 [2.53, 3.28]	2.75 +/− .078 [2.59, 3.28]
ONB M +/− SD *range*	2.88 +/− .089 [2.58, 3.46]	2.90 +/− .093 [2.59, 3.46]	2.92 +/− .095 [2.70, 3.46]
OCL M +/− SD *range*	1.00 +/− .137 [.223, 1.57]	.95 +/− .139 [.223, 1.57]	.930 +/− .146 [.432, 1.41]

**Table 2.** Linear mixed-effects regression parameter estimates (standard errors) for predicting CBB performance

	DET RT		IDN RT		ONB RT		OCL accuracy	
	b (SE)	p	b (SE)	p	b (SE)	p	b (SE)	p
Time	−.006 (.004)	.13	−.005 (.002)	<.05	−.009 (.003)	<.01	.041 (.006)	<.001
Baseline age	.003 (.0001)	<.001	.002 (.00007)	<.001	.003 (.0001)	<.001	−.002 (.0001)	<.001
Gender (0 = male, 1 = female)	.026 (.001)	<.001	.002 (.001)	<.05	.006 (.001)	<.001	.012 (.002)	<.001
Years education	−.003 (.0003)	<.001	−.001 (.0002)	<.001	−.002 (.0003)	<.001	.006 (.0004)	<.001
Ethnicity (0 = non-Caucasian, 1 = Caucasian)	−.024 (.002)	<.001	−.014 (.001)	<.001	−.019 (.002)	<.001	.023 (.003)	<.001
Family history of AD (0 = no, 1 = yes)	.0005 (.001)	.74	.0002 (.001)	.78	.004 (.001)	<.01	.0008 (.002)	.68
Self-reported AD (0 = no, 1 = yes)	.030 (.005)	<.001	.022 (.004)	<.001	.022 (.005)	<.001	−.042 (.007)	<.001
Self-reported MCI (0 = no, 1 = yes))	.027 (.002)	<.001	.016 (.001)	<.001	.020 (.002)	<.001	−.023 (.003)	<.001
Subjective memory concern (0 = no, 1 = yes)	.008 (.001)	<.001	.008 (.001)	<.001	.011 (.001)	<.001	−.014 (.002)	.68
Time × Baseline age	.00004 (.00004)	.36	.00002 (.00003)	.44	.00003 (.00004)	.44	−.0003 (.00007)	<.001
Time × Gender	−.0009 (.0006)	.15	−.0007 (.0004)	.07	−.00008 (.0005)	.87	.0007 (.001)	.48
Time × Years education	−.0001 (.0001)	.47	.00004 (.00007)	.59	−.00009 (.0001)	.38	−.00006 (.0002)	.74
Time × Ethnicity	.0003 (.0009)	.74	.001 (.0006)	.10	.001 (.0008)	.09	−.0008 (.002)	.60
Time × Self-reported MCI	.0009 (.0009)	.31	.0004 (.0005)	.45	.002 (.0007)	<.05	−.005 (.001)	<.001
Time × Subjective Memory concern	.002 (.0006)	<.05	−.00001 (.0004)	.98	.0005 (.0005)	.32	−.001 (.001)	.28
Time × Self-reported AD	.0002 (.002)	.95	.000003	.99	−.0004 (.002)	.82	.005 (.004)	.22
Time × Family history of AD	−.0006 (.0006)	.30	−.0005 (.0004)	.18	−.001 (.0005)	<.05	−.0009 (.0009)	.35

**Table 3.** Logistic regression odds ratios for predicting performance decline on CBB subtests

	DET RT (N = 943)	IDN RT (N = 940)	ONB RT (N = 934)	OCL accuracy (N = 961)
	OR (95% CI)	OR (95% CI)	OR (95% CI)	OR (95% CI)
Baseline age	1.01* (1.00, 1.02)	1.00 (.99, 1.01)	1.01 (.99, 1.02)	1.00 (.99, 1.01)
Gender (0 = male, 1 = female)	1.08 (.93, 1.25)	1.04 (.90, 1.21)	.99 (.86, 1.15)	1.02 (.88, 1.18)
Years education	1.01 (.98, 1.04)	1.05* (1.02, 1.08)	1.05 (1.02, 1.08)	1.06 (1.03, 1.09)
Ethnicity (0 = non-Caucasian, 1 = Caucasian)	1.06 (.87, 1.30)	1.04 (.85, 1.27)	1.26* (1.02, 1.57)	1.38* (1.12, 1.73)
Family history of AD (0 = no, 1 = yes)	1.08 (.94, 1.24)	.93 (.81, 1.07)	1.04 (.90, 1.19)	.98 (.85, 1.12)
Self-reported AD (0 = no, 1 = yes)	.78 (.48, 1.20)	.76 (.45, 1.21)	.69 (.38, 1.14)	.95 (.57, 1.49)
Self-reported MCI (0 = no, 1 = yes))	1.63* (1.35, 1.95)	1.45* (1.20, 1.76)	1.26* (1.02, 1.57)	1.18 (.96, 1.44)
Subjective memory concern (0 = no, 1 = yes)	1.15* (1.00, 1.34)	1.13 (.98, 1.31)	1.26* (1.09, 1.47)	1.12 (.97, 1.30)

* p < .05
